# Proposal for a Quantitative ^18^F-FDG PET/CT Metabolic Parameter to Assess the Intensity of Bone Involvement in Multiple Myeloma

**DOI:** 10.1038/s41598-019-52740-2

**Published:** 2019-11-11

**Authors:** Maria E. S. Takahashi, Camila Mosci, Edna M. Souza, Sérgio Q. Brunetto, Elba Etchebehere, Allan O. Santos, Mariana R. Camacho, Eliana Miranda, Mariana C. L. Lima, Barbara J. Amorim, Carmino de Souza, Fernando V. Pericole, Irene Lorand-Metze, Celso D. Ramos

**Affiliations:** 1 School of Medical Sciences, University of Campinas, Campinas, Brazil; 2 Gleb Wataghin Physics Institute, University of Campinas, Campinas, Brazil; 3 Division of Nuclear Medicine, University of Campinas, Campinas, Brazil; 4 Center of Biomedical Engineering, University of Campinas, Campinas, Brazil; 5 Center of Hematology and Hemotherapy, University of Campinas, Campinas, Brazil; 6 Department of Internal Medicine, Faculty of Medical Sciences, University of Campinas, Campinas, Brazil

**Keywords:** Radionuclide imaging, Cancer imaging

## Abstract

Many efforts have been made to standardize the interpretation of ^18^F-FDG PET/CT in multiple myeloma (MM) with qualitative visual analysis or with quantitative metabolic parameters using various methods for lesion segmentation of PET images. The aim of this study was to propose a quantitative method for bone and bone marrow evaluation of ^18^F-FDG PET/CT considering the extent and intensity of bone ^18^F-FDG uptake: Intensity of Bone Involvement (IBI). Whole body ^18^F-FDG PET/CT of 59 consecutive MM patients were evaluated. Compact bone tissue was segmented in PET images using a global threshold for HU of the registered CT image. A whole skeleton mask was created and the percentage of its volume with ^18^F-FDG uptake above hepatic uptake was calculated (Percentage of Bone Involvement - PBI). IBI was defined by multiplying PBI by mean SUV above hepatic uptake. IBI was compared with visual analysis performed by two experienced nuclear medicine physicians. IBI calculation was feasible in all images (range:0.00–1.35). Visual analysis categorized PET exams into three groups (negative/mild, moderate and marked bone involvement), that had different ranges of IBI (multi comparison analysis, p < 0.0001). There was an inverse correlation between the patients’ hemoglobin values and IBI (r = −0.248;p = 0.02). IBI score is an objective measure of bone and bone marrow involvement in MM, allowing the categorization of patients in different degrees of aggressiveness of the bone disease. The next step is to validate IBI in a larger group of patients, before and after treatment and in a multicentre setting.

## Introduction

Lytic bone lesions are reported in approximately 80% of myeloma multiple (MM) patients^1,2^. Early and precise evaluation of bone involvement is crucial for staging and correct disease management.

Hybrid image of positron emission tomography with ^18^F-fluordeoxyglucose and computed tomography (^18^F-FDG PET/CT) is one of the main methods for the evaluation of MM patients. It allows whole-body images, intra and extramedullary lesion detection, distinction between active lesions and scar or necrotic tissue and has been more sensitive than MRI in treatment assessment^3–5^.

Many efforts have been attempted to standardize the interpretation of ^18^F-FDG PET/CT in MM, using qualitative visual analysis or quantitative metabolic parameters, such as metabolic tumor volume (MTV) and total lesion glycolysis (TLG)^6–9^. However, none of these methods have been extensively used in clinical practice or research projects, probably because of the complexity of the visual quantification^6,7^ or due to the lack of standardization of MTV and TLG calculations^8–10^. Also, MTV and TLG only consider areas visually defined as lesions and ignore diffuse uptake of the bone marrow.

Here, we propose a semi-automatic method to obtain a quantitative parameter for metabolic activity of the bone affected by MM in ^18^F-FDG PET/CT images, defined as Intensity of Bone Involvement (IBI). For this, CT-based bone segmentation is critical to obtain a standardized and reproducible quantitative assessment of bone ^18^F-FDG uptake, since the direct segmentation of PET images is difficult to standardize, especially in cases of diffuse involvement.

## Materials and Methods

**Figure 1:**
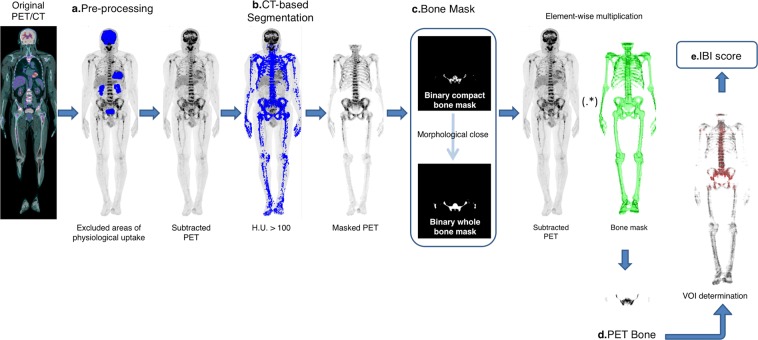
Five major steps for Intensity of Bone Involvement (IBI) calculation. (**a**) Original PET was pre-processed to clean the high intensity areas of physiological uptake and external objects. (**b**) Then, the image was segmented according to the Hounsfield scale (HU) of the registered CT. In this step, the skull region had to be excluded from the mask to reduce the artifacts caused by the overlapping of brain uptake (for patients with focal lesions on skull, a manual correction for IBI can be performed, see text). (**c**) Masked PET was transformed into a binary matrix and a morphological close was performed to achieve the final bone mask. (**d**) Multiplication between bone mask and subtracted PET resulted in the PET image containing only the bone and bone marrow tissues (PETbone/bm). (**e**) Metabolic metrics, including IBI score, were performed on PETbone/bm imaging.

IBI calculation was performed in five major steps: pre-processing, CT-based segmentation, bone mask creation, creation of a PET image containing only the bone and bone marrow tissues (PETbone/bm) and metabolic metrics calculation. Figure 1 shows an overview of the process used for IBI calculation.

### Image pre-processing and CT-based segmentation

Image pre-processing was performed to reduce the influence of artifacts on metabolic results. It included manual extraction of external objects (e.g., patient table and urinary catheter) and subtraction of high intensity areas of physiological uptake (heart, kidneys, bladder, etc) through auto-segmentation of the PET image. The “clean” PET was called subtracted PET.

A CT-based segmentation was made using global thresholding^11–14^. In this case, Hounsfield index (HU) higher than 100 was used as a criterion to segment the whole compact bone in the CT image. All voxels below this value was set to zero.

A Masked PET was then obtained, and it corresponded to anatomical contour of compact bone in the functional image. In this step, the skull region had to be excluded from the mask because the pre-processing was not enough to reduce the artifacts caused by the overlapping of brain uptake. In patients with focal skull lesions, a manual correction for IBI calculation was performed  (see bellow).

### Creation of bone mask and “PET bone/bone marrow”

Bone marrow has the same HU range as soft tissues and for this reason it is not included when CT-based segmentation is performed. To include the bone marrow in the masked region, the Masked PET was turned into a binary image and a morphological close operation was performed using a disk-shaped structuring element with radius of 3 pixels. The result was a binary bone mask that included also the medullary cavity.

Element-wise multiplication of bone mask and subtracted PET was performed. Since the bone mask was a binary matrix, everything that is outside the mask was set to 0. The final result was a segmented PET image that shows only bone and bone marrow uptake (“PET bone/bone marrow” or PETbone/bm).

### Bone/bone marrow metabolic metrics

PETbone/bm is the foundation image to obtain the metabolic metrics of bone and bone marrow tissue for MM patients. Three basic metabolic metrics where directly extracted from the images: maximum SUV of bone tissue (SUV_max_), mean SUV of bone tissue (SUV_mean_) and standard deviation of bone tissue SUV (σ).

A volume of interest was defined (VOI) as the volume whose ^18^F-FDG uptake is above the mean liver uptake plus two standard deviation ($\overline{SU{V}_{liver}}+2{\sigma }_{liver}$); this is the volume considered to be metabolically active. This VOI comprises any voxel within this criterion, even if it is isolated in an area with lower ^18^F-FDG uptake.

We defined as percentage of bone involvement (PBI) the fraction of segmented bone tissue whose ^18^F-FDG uptake is above the liver uptake. PBI is calculated as the division of VOI by the total bone mask volume (BMV), Eq. (1).

1 $$PBI=\frac{VOI}{BMV}$$

The other metabolic parameter proposed is the Intensity of Bone Involvement (IBI). IBI is calculated as the multiplication of PBI and mean SUV of the VOI, Eq. (2).

2 $$IBI=PBIx\overline{SU{V}_{VOI}}$$

This quantity takes into account the extent and metabolic intensity of the bone uptake, similarly to TLG^15^, except in two aspects. First, it is not based on an absolute volume but on a fraction of volume and second, diffuse pattern of ^18^F-FDG uptake is always included in the calculation.

For patients with focal lesions in the skull, a manual correction for IBI calculation can be performed. For a patient with “n” focal skull lesions, IBI becomes

3 $$IBI=PBIx\overline{SU{V}_{VOI}}+\,(\frac{{\sum }_{i}^{n}SL{V}_{i}\times {\overline{SUV}}_{i}}{BMV})$$

where SLV_i_ is the volume of the *i-th* skull lesion manually determined and ${\overline{SUV}}_{i}$ is its respective mean SUV.

The mean and standard deviation of hepatic SUV of the 59 patients were calculated to assess the variability of this parameter in these patients.

### Patient evaluation using IBI

We calculated IBI for whole-body ^18^F-FDG PET/CT images of 59 consecutive patients diagnosed with MM before or at the beginning of their treatment. Patients were diagnosed according to the International Myeloma Working Group (IMWG) 2014^16^, including bone marrow cytology and histology. This retrospective study was approved by the University of Campinas Ethics Committee (Registration Number: CAAE 97966618.5.0000.5404). The need for written informed consent was waived by the Ethics Committee.

The patients were instructed to fast for at least 6 hours. All patients were scanned from head to feet, according to the standard protocol for MM of our center. Image acquisitions started 60 min after the injection of 0.12 mCi/kg of ^18^F-FDG, in a Biography mCT40 PET/CT scanner (Siemens Medical, USA). The CT part of the study was acquired with 120–140 kV, 120 mA, transaxial FOV 700 mm, rotation time 0.8 s, and slice thickness 2.1 mm. The emission scan was performed in a 3D mode, 1.5 min per bed position. PET images were reconstructed using a standard iterative algorithm (3D-OSEM + PSF+TOF with 2 iterations and 21 subsets), with the CT data utilized for attenuation correction and image fusion.

A spherical VOI of 34,69 cm^3^ (radius ∼2,0 cm) was placed in the liver of each image to find the mean and standard deviation of the background SUV, used as threshold for IBI calculation.

Pre-processing, CT-based segmentation and liver SUV were performed using the Beth Israel Plugin for FIJI^17,18^. IBI, as well as final bone mask and PETbone/bm, was performed with an in-house software implemented in MATLAB^19,20^.

The following clinical and laboratory parameters were obtained from patients: stage of disease according to the International Stage System (ISS), hemoglobin, percentage of plasma cell of bone marrow (BM), lactate dehydrogenaze (LDH), serum calcium and creatinine. Patients’ characteristics are described in Table 1.

**Table 1:** Patient’s characteristics.

No. of patients	59
female	28 (47.5%)
male	31 (52.5%)
Age (y)	
Mean ± SD	64.2 ± 12.2
Range	36.8–87.2
ISS	
I	13 (22.0%)
II	8 (13.6%)
III	38 (64.4%)
Anaemia (hemoglobin <10.0 g/dl)	32 (54.2%)
Plasma Cell (BM) >20%	37 (62.7%)
Hypercalcaemia	10 (16.9%)
Renal Insufficiency	21 (35.6%)
Soft tissue involvement	27 (45.8%)
Extramedullary disease	4 (6.8%)

### Visual analysis versus IBI score

Visual analysis of all whole-body ^18^F-FDG PET/CT images was performed by two experienced nuclear medicine physicians. The criteria to consider ^18^F-FDG PET/CT as “positive” for MM bone involvement was the presence of hypermetabolic focal bone lesions and/or diffuse increased uptake in the bone marrow.

The criteria used to classify the intensity of bone involvement of the “positive” images was based in the Deauville score routinely utilized for the evaluation of ^18^F-FDG uptake in lymphomas, and used the liver as reference^21^. Lesion ^18^F-FDG uptake lower or similar to liver uptake, moderately higher or markedly higher than the liver uptake, were respectively classified as mild, moderate or marked bone involvement.

The number of focal lesions on ^18^F-FDG PET/CT image of each patient was also evaluated by the nuclear medicine physicians. Images were classified into four groups: no focal lesions, 1 to 3 focal lesions, 4 to 10 focal lesions and more than 10 focal lesions.

## Results

**Figure 2:**
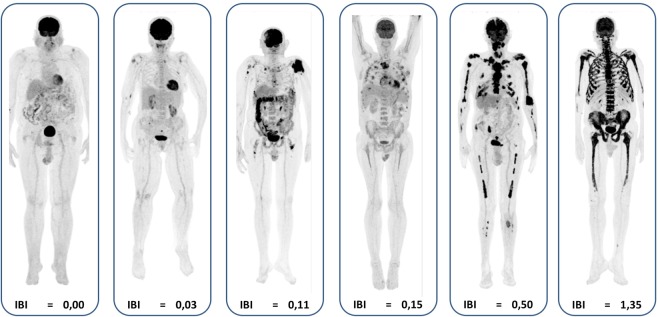
Maximum instensity projection (MIP) of PET images of six different patients progressively aligned from left to right, according to the extent of bone involvement, as stated by a subjective visual analysis of PET images: negative/mild (the two images on the left), moderate (the two central images) and marked bone involvement (the two images on the right). Note that the Intensity of Bone Involvement (IBI) score increases progressively, so that the bone/bone marrow involvement can be evaluated as a continuous numerical variable.

IBI calculation was feasible in all 59 ^18^F-FDG PET/CT images and ranged from 0.00 to 1.35 (Fig. 2). Twenty-nine of the 59 PET exams were visually classified as negative or with mild bone involvement, 16 presented moderate and 14 had marked bone involvement (Table 2). A multi comparison analysis showed that the median IBI score was different in each of the three groups (p-value < 0.0001, Kruskal-Wallis with Dunn´s post-hoc test).

**Table 2:** IBI scores for the three groups of visual classification of bone involvement.

*Visual Classification of bone involvement*	*n*	*Median*	*Range*
Negative/mild	29	0.02	(0.00–0.09)
Moderate	16	0.05	(0.01–0.15)
Marked	14	0.26	(0.07–1.35)

All of the 59 patients could be classified according to the number of focal lesions in the initial staging ^18^F-FDG PET/CT. Eleven patients had no focal lesions identified in the initial ^18^F-FDG PET/CT image, ten patients had 1 to 3 focal lesions in the initial ^18^F-FDG PET/CT, sixteen patients had 4 to 10 focal lesions in the initial ^18^F-FDG PET/CT and twenty-two patients were classified as having more than 10 focal lesions in the initial ^18^F-FDG PET/CT **(**Table 3). Significant differences in the IBI scores were found between the group of patients with more than 10 focal lesions and the groups with 1–3 and 4–10 lesions (p-value < 0.05).

**Table 3:** Intensity of Bone Involvement (IBI) scores for the four group of patients classified by the number of focal lesions.

*Number of focal lesions*	*n*	*Median of IBI scores*	*Range of IBI scores*
0	11	0.02	(0.01–0.60)
1–3	10	0.02	(0.00–0.15)
4–10	16	0.02	(0.00–0.50)
>10	22	0.11	(0.02–1.35)

There was an inverse correlation between the hemoglobin values of the patients measured at the time of PET and IBI (r = −0.248; p = 0.02) and PBI(r = −0.264; p = 0.043) but not with $\overline{SU{V}_{VOI}}$ (r = 0.13; p = 0.14).

Focal skull lesions were found in three of 59 PET images. The initial IBI values of these patients (without correction for skull region exclusion) were 0.07, 0.41 and 0.50. After manual correction for the skull lesions, IBI values were respectively 0.08, 0.42 and 0.50.

The mean hepatic SUV in our group of 59 patients was 2.21 and the standard deviation 0.44. The percentiles 10–90% were 1.66–2.74. No focal lesions were identified in any of the patients. No patient had diffuse liver involvement detectable by FDG-PET/CT.

### Technical limitations of IBI calculation

**Figure 3:**
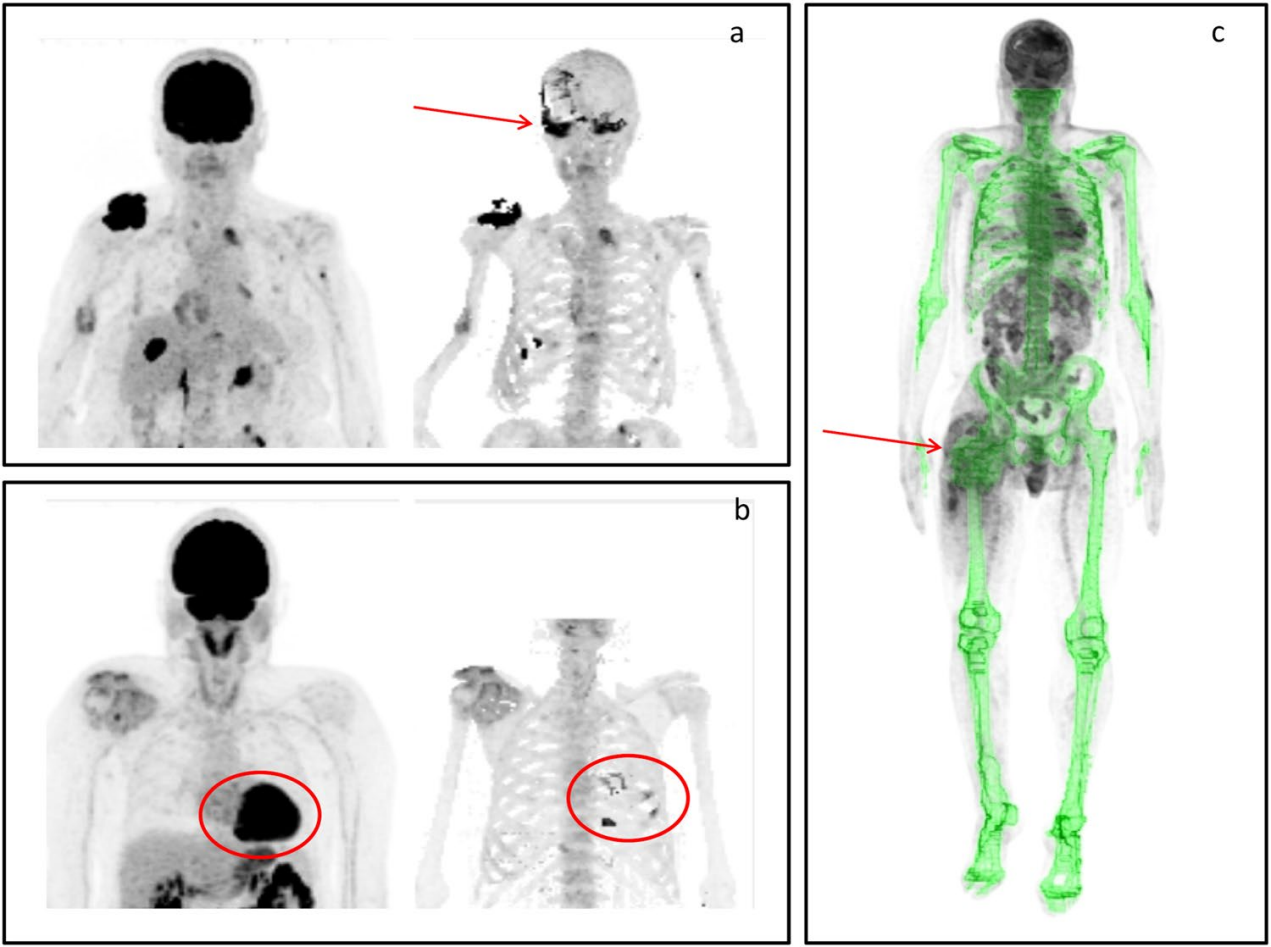
Artifacts that may influence Intensity of Bone Involvement score calculation. (**a**) Original (left) and processed (right) images: note the brain uptake overlapping the skull (arrow). (**b**) Heart uptake overlapping the ribs (circumferences on the original and processed images). (**c**) Bone mask contour influenced by a metallic femoral prosthesis (arrow).

Some artifacts were observed during IBI calculation. The most recurrent of them was the overlapping of brain ^18^F-FDG uptake in the masked PET image. For this reason, the subtraction of skull for IBI and PBI calculation seemed to be a reasonable approximation. This can be manually corrected when focal skull lesions are present. Other areas of physiologic uptake, such as heart and bladder, also overlapped compact bone areas in some patients, even after subtraction of auto-segmented areas on FIJI (Fig. 3a,b). Metallic implants, such as femoral prostheses, distorted the natural contour of the skeleton in the bone mask (Fig. 3c).

## Discussion

When analyzing ^18^F-FDG PET/CT images of MM patients, it is usually not difficult to identify extra-osseous lesions, just as in the evaluation of solid tumors. In contrast, it is sometimes very challenging to classify bone marrow involvement in the PET scans of many of these patients, because both focal and diffuse bone lesions may coexist in MM, with varying degrees of ^18^F-FDG uptake. The IBI score objectively addresses this issue.

Several methods for quantifying the extent of disease in MM patients have been proposed in recent years. In 2016, Nanni *et al*.^7^ described a visual method of ^18^F-FDG PET/CT interpretation (IMPeTUs) where each lesion is individually analyzed using the Deauville five point-scale^21^ or by four points scoring system. Although this method is very comprehensive and considers different types of osseous and extra-osseous sites of lesions, including diffuse bone pattern, it is not practical in daily routine and does not provide a general parameter that represents the degree of whole bone involvement. The simple visual analysis is a practical alternative. However, it is somewhat subjective and depends on the observer’s experience. Therefore, it is of limited reproducibility among different centers.

MTV and TLG have also been used as promising metabolic parameters to quantify lesions in oncology, including MM^8,9,22–25^. However, segmentation methods for MTV determination are not yet standardized which prevents comparisons between studies. Most techniques use a fixed or a percentage threshold, which are simple to use but have some disadvantages, such as not taking the background activity into account. More specifically, MTV calculated by percentage threshold has a low sensitivity because it does not always cover the whole tumor volume and has a strong dependence of the scanner sensitivity and reconstruction methods^10,26^.

Compared to TLG, IBI considers any voxels above the threshold, not only lesion-like areas. Moreover, IBI does not use an absolute volume, but a ratio between metabolic volume above hepatic uptake and bone/bone marrow mask volume with the purpose of weighing the score by the skeletal size of each patient. Using liver SUV as reference, it is possible to partially compensate for factors that influence SUV, such as patient weight. Several authors have used this approach to analyze different diseases^10,21,27^, including MM^6,7^, because the liver is one of the organs with the most constant FDG uptake^28^.

Since normal bone marrow FDG uptake is generally lower than hepatic uptake, using the liver as an internal reference allowed us to highlight the metabolic activity of the disease in IBI. This is because including “normal uptake” (below hepatic) in IBI would reduce the differences between the several degrees of MM involvement. For example, mild and moderate bone involvement would have relatively close numerical values if we added the “normal uptake” value to both.

The central idea of the IBI score is to provide a consistent and objective numerical variable for grading the intensity of bone marrow involvement in MM patients, using a measure related to an internal control (liver), both at the initial evaluation as well as for assessment of response to treatment. Bone segmentation through the HU scale of CT images is crucial for the reproducibility of the proposed parameter, making it less operator-dependent than methods that use only the PET images for segmentation.

Global thresholding method is one of the most used methods for bone segmentation and it is available in many commercial software for image processing. Although other methods have shown to be more accurate, like convolutional neural network (CNN)^29^, PET image does not have enough spatial resolution that rewards the extra time needed for this kind of bone segmentation.

We found that IBI score had a good agreement with the subjective analysis of the extent of bone involvement in ^18^F-FDG PET/CT images. High values of IBI were related to a more extensive and/or intense ^18^F-FDG uptake, allowing patients to be graded according to the intensity of bone involvement. Interestingly, the number of focal lesions had a poor agreement with the IBI score, mainly when no focal lesions were identified on the ^18^F-FDG PET/CT images. That could be expected since just counting the number of lesions does not consider the presence of diffuse bone marrow involvement, which is included in the IBI calculation.

We found an inverse correlation between hemoglobin values and IBI. Although anemia has a complex pathophysiology in MM, this is probably because IBI is higher in patients with more extensive disease. The most common cause is the anemia of chronic disease, characterized by an inhibition of erythropoiesis, impaired iron metabolism and up regulation of hepcidin m-RNA^30^. Besides, anemia can also be due to chronic renal insufficiency, and more rarely by hemolysis or myelodysplasia.

Like different other metabolic parameters, IBI is SUV-dependent. Therefore, every factor affecting SUV value will also affect IBI results: reconstruction and acquisition parameters, partial-volume correction, blood glucose, time between ^18^F-FDG injection and image acquisition etc.^31–33^. Hence, SUV harmonization is essential for comparing results among patients, for the same patient in its follow-up or in multicenter trials. On the other hand, since IBI utilizes the hepatic SUV as a reference and a large bone volume for calculation (the skeletal mask), the influence of SUV is probably less than for other techniques that use a fixed SUV threshold for lesion segmentation.

The methodology described here could potentially be adapted to compare different radiotracers recently proposed for evaluating MM, such as ^11^C-choline^34^, ^11^C-methionine^34^, ^18^F-fluoro-ethyl-tyrosine^35^, ^68^Ga-DOTATATE^36^ and ^68^Ga-PSMA^37^. MM lesions frequently present varying degrees of uptake of those radiotracers compared to FDG uptake^35,36^, probably due to the extensive intra and inter-patient genomic heterogeneity of the disease^38^. An objective quantitative comparison of those tracers in MM might be of interest. An adapted IBI methodology could also be used to quantify ^18^F-NaF PET/CT, especially when evaluating disseminated osteoblastic lesions, such prostate cancer metastases.

High ^18^F-FDG uptake of the brain and limited spatial resolution of PET images taken together create artifacts that hamper the inclusion of skull in IBI calculation, and focal lesions in this area must be analyzed independently. Although skull involvement is relatively common in MM, the bone sites most frequently and extensively affected by the disease are the spine, pelvis, sternum and proximal metaphyses of long bones, because adult bone marrow is predominantly confined in these sites^39,40^. In our sample of 59 patients, only three (5%) had cranial involvement, demonstrated by ^18^F-FDG-PET/CT, all with relatively small lesions compared to those of the spine and pelvis. We manually included the skull lesions in IBI calculation of these patients, even though we found no important changes in IBI values after this correction, probably due to the small size of these lesions. Albeit making the method more operator-dependent, this manual option can be used in selected cases with extensive cranial involvement or other imaging artifacts.

Liver activity may vary from subject to subject. While useful as reference for normalizing factors that interfere with SUV, e.g., body weight and blood glucose, liver activity can be an error source if the underlying disease secondarily involves this organ. Although clinical manifestations of liver involvement by MM are rare^41^, liver abnormalities is reported to be relatively common in autopsy series, including amyloidosis, light-chain deposition disease, extramedullary plasmacytomas, and diffuse infiltrative process^41^. When clinically relevant, these processes should cause an increase in liver FDG uptake, as suggested by very few reports of both focal lesions^42^ and diffuse involvement^43^. That was not the case in our patients, since no focal liver lesion or diffuse involvement was detected. We found a mean liver SUV of 2.21 with a standard deviation of only 0.44 in these patients.

On the other hand, if diffuse liver involvement is detected on FDG-PET images or if there is clinical suspicion of relevant liver involvement, a reported fixed SUV threshold of 2.5^8,24^ should be considered instead of using the liver as reference. The mean liver SUV of 2.21 found in our MM patients with no known liver disease could also be used as fixed threshold. In cases of focal lesions, the operator should simply avoid overlap them when drawing the reference liver ROI.

Although we had no cases in which lytic lesions were excluded in the proposed method for bone masking, it is theoretically possible that very extensive lytic lesions could not be incorporated by the bone mask, since the HU scale of that region would be very different from a healthy bone tissue. In these cases, manual contour corrections of the masks should be performed. Obviously, IBI does not evaluate isolated extra-osseous MM lesions and, when present, they should be analyzed separately.

At a first glance, the method described here has some complexity, but the image processing steps used for PBI and IBI calculation are available on many commercial workstations and image software (including some free ones, such as FIJI). Basically, we used global threshold segmentation, morphological operations and matrix multiplication, which are simple operations compared to machine and deep learning, for example. We believe it can be an accessible method in terms of operational complexity and feasible in clinical practice.

## Conclusion

The proposed IBI score is an objective measure of bone marrow lesions in patients with MM with different degrees of bone involvement. It may allow comparisons among patients in multicenter settings and measurements before and after treatment. It seems to be a feasible parameter for use in clinical practice and research. Further studies are needed to evaluate the possible clinical role of IBI in patients with MM.

## Data Availability

The datasets generated during and/or analyzed during the current study are not publicly available due to protect the identity of research subjects but are available from the corresponding author on reasonable request.
